# Association of leisure-time aerobic physical activity time with apparent treatment-resistant hypertension: a study based on NHANES database

**DOI:** 10.5114/biolsport.2025.148543

**Published:** 2025-04-14

**Authors:** Wanling Huang, Dongqin Lai, Meie Zeng, Bin Chen, Shuifen Ye, Fenjing Li, Chunmei Huang

**Affiliations:** 1 General Practice Department, Longyan First Affiliated Hospital of Fujian Medical University, Longyan City, Fujian Province, 364000, China

**Keywords:** Apparent treatment-resistant hypertension, Leisure-time aerobic physical activity, NHANES, Risk

## Abstract

Using information from the National Health and Nutrition Examination Survey (NHANES) database, this study intends to investigate the connection between apparent treatment-resistant hypertension (aTRH) and leisure-time aerobic physical activity (LTAPA) time. A cross-sectional analysis was conducted on the NHANES data from 2007 to 2018. LTAPA time was used as the independent variable, and aTRH was used as the dependent variable. Participants’ baseline characteristics were collected and analyzed based on whether they had aTRH. A weighted regression model was used to analyze association of LTAPA time with aTRH, and a stratified analysis was conducted. In addition, the relationship between LTAPA and the risk of all-cause mortality in patients with aTRH was assessed and survival curves were plotted. 6,705 hypertensive patients were included, with 6,292 in non-aTRH group and 413 in aTRH group. Baseline results presented significant differences in age, race, povertyto-income ratio (PIR), body mass index (BMI), number of cases of diabetes mellitus, and estimated glomerular filtration rate (eGFR) values between two groups (p < 0.05). Weekly LTAPA time was significantly lower in aTRH group than in non-aTRH patients (76.15 vs. 103.16, p = 0.016). Weighted logistic regression modeling showed that an increase in LTAPA time was associated with a reduced risk of developing aTRH (OR = 0.939, 95%CI [0.884,0.999], p = 0.045), especially among female patients who did not drink alcohol. Weighted Cox regression analysis showed a significant negative association between LTAPA time and risk of all-cause mortality (OR = 0.900, 95%CI [0.831,0.976], p = 0.010). Kaplan-Meier survival analysis also showed that an increase in LTAPA time significantly prolonged the survival time of aTRH patients (Log-rank p < 0.001). Increasing LTAPA time among American adults was associated with the reduced incidence and mortality of aTRH, especially in women who do not drink alcohol. In the future, gender-specific exercise strategies could be considered, along with drug therapy and other non-pharmacological interventions, to develop targeted treatment strategies for hypertensive patients to reduce the incidence and mortality of aTRH.

## INTRODUCTION

Hypertension is a common preventable risk factor that affects the incidence and mortality of cardiovascular disease (CVD) – related diseases and premature deaths globally and is a risk factor for many adverse cardiovascular outcomes, including peripheral or coronary artery disease, heart failure, myocardial infarction, and stroke [1–3]. One of the global objectives for non-communicable illnesses is to lower the prevalence of hypertension by 33% between 2010 and 2030, with an estimated 1.28 billion people globally suffering from hypertension [4]. Resistant hypertension (RH) is characterized by blood pressure that remains uncontrolled (systolic ≥ 140 mmHg or diastolic ≥ 90 mmHg) even after taking at least three antihypertensive drugs, or at least four antihypertensive drugs before blood pressure is brought under control [5]. Apparent treatment-resistant hypertension (aTRH) is the most common term in the clinic and in many studies, and the term is used because of the inability to rule out inaccurate blood pressure measurements, medication nonadherence leading to elevated blood pressure, or white coat effect (in-office blood pressure above target, but out-of-office blood pressure equal to or below the target value) and other pseudo-RH [6–8]. In 2018, American Heart Association’s scientific statement on aTRH stated that the prevalence of aTRH is 19.7%, or approximately 10.3 million U.S. adults with aTRH [9]. Regardless of blood pressure control, people with aTRH have an increased risk of coronary artery disease, heart failure, and other CVD, end-stage renal disease, and all-cause mortality [5, 10, 11]. aTRH is undoubtedly an important public health problem and therefore there is an urgent need to develop appropriate prevention strategies to avoid the development of lifethreatening diseases.

Clinical management of patients at any stage of hypertension cannot be separated from healthy lifestyle and pharmacologic interventions. The four major classes of drugs for hypertension include renin-angiotensin-aldosterone system (RAAS) blockers, beta blockers, calcium channel blockers, and thiazide or thiazide diuretics. Pharmacologic treatment significantly improves morbidity and mortality in hypertensive patients with CVD [12]. But in a combined analysis including 104 million individuals, it was found that less than half of people with hypertension received medication and only 23% of women and 18% of men ended up with controlled blood pressure [13]. Therefore, many clinical guidelines for hypertension worldwide suggest the use of non-pharmacological interventions throughout the entire course of hypertension treatment. A few examples of non-pharmacological therapies are cutting back on salt, eating more fruits and vegetables, exercising, giving up smoking, and switching to a low-fat dairy product [14, 15].

Exercise has been widely shown to have antihypertensive effects [16–18]. Strong evidence suggests that engaging in moderate to high-intensity physical activities, especially aerobic exercise, can improve cardiorespiratory health and reduce incidence of hypertension [19]. Leisure-time aerobic physical activity (LTAPA) refers to aerobic behaviors that people engage in during their leisure time, including walking, running, biking, and soccer [20]. These exercises have been extensively studied and have been shown to have a positive effect on lowering blood pressure. Results from relevant randomized controlled trials suggest that leisure-time aerobic physical activities such as walking, soccer, and swimming can lower blood pressure levels in patients with hypertension [21–23]. Additionally, a randomized controlled trial study on the cardiovascular effects of physical exercise on resistant hypertension revealed that physical exercise could lower blood pressure even in subjects with low response to drug therapy [24].

However, most of these studies were small sample trials and failed to adequately explore the association between LTAPA and aTRH. There is a lack of studies based on large sample data to assess the relationship between LTAPA and aTRH. Therefore, this study aimed to investigate the association between LTAPA and aTRH incidence and mortality through a large sample of clinical data from the National Health and Nutrition Survey (NHANES), so as to provide policymakers with a scientific basis for the prevention and treatment of aTRH.

## MATERIALS AND METHODS

### Data Source and Study Population

In order to evaluate the health and nutritional status of American adults and children, the National Center for Health Statistics (NCHS) of the United States conducted a study project called NHANES. The project used a sophisticated, multi-stage probabilistic sampling method to draw samples from the non-institutionalized civilian population of the United States. The NCHS Research Ethics Review Board approved the NHANES study protocol, and informed consent forms were signed by each participant. Relevant information can be accessed for free on the website (http://www.cdc.gov/nchs/nhanes.htm).

**FIG. 1:**
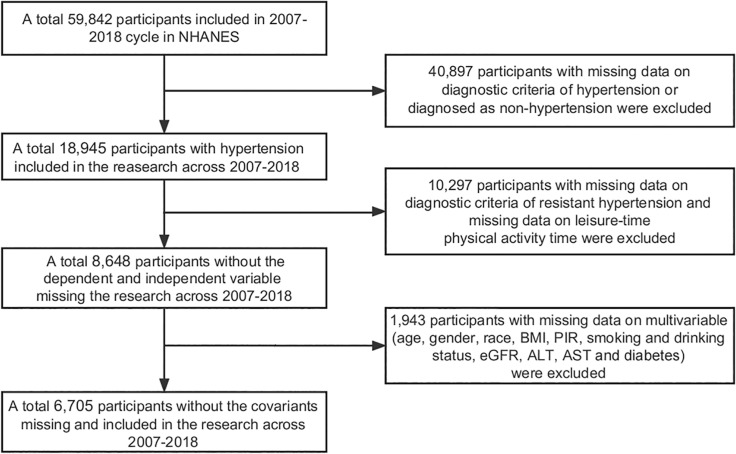
NHANES 2007–2018 Sample Selection Flowchart.

We utilized data from six cycles of NHANES spanning from 2007 to 2018 for this study. During this survey period, data were collected from 59,842 individuals. We excluded participants with missing data on hypertension diagnosis and non-hypertensive individuals (40,897), as well as participants with missing data on aTRH diagnosis and LTAPA (10,297). In addition, participants with missing data on multiple variables (age, gender, race, body mass index (BMI), aspartate aminotransferase (AST), smoking and alcohol drinking, estimated glomerular filtration rate (eGFR), poverty-to-income ratio (PIR), alanine aminotransferase (ALT), and diabetes) were also excluded (1,943). Finally, data from 6,705 participants were included, and the specific participant selection process is shown in Figure 1.

### Leisure-Time Aerobic Physical Activity

The independent variable in this study was LTAPA, which was assessed in participants through the following two questions: (1) “Did you engage in at least 10 minutes of moderate-intensity activity in the last 30 days, such as brisk walking, cycling, golfing, or dancing, that only resulted in light perspiration or a small to moderate rise in respiration or heart rate?” (2) “During the past 30 days, did you perform at least 10 minutes of high-intensity exercise that caused substantial sweating or a large increase in breathing or heart rate, such as running, swimming, aerobics classes, or fast biking?” Participants who answered “yes” to the above questions were further asked about the specific type of exercise, the frequency of exercise per week, and the average duration of each exercise session [25]. Weekly LTAPA time was calculated using the following formula:

LTAPA Time Per Week = High Intensity LTAPA Per Day * High Intensity LTAPA Days Per Week * 2 + Moderate Intensity LTAPA Per Day * Moderate Intensity LTAPA Days Per Week [25].

According to the 2008 guidelines [26], the amount of LTAPA in this study was categorized into 3 classes: (1) exercise was sufficient if participants reported ≥ 150 minutes/week of moderate-intensity exercise, or ≥ 75 minutes/week of high-intensity exercise or an equivalent combination of ≥ 150 minutes/week; (2) if participants reported some exercise but not enough to meet the definition of adequate exercise (> 0 to < 150 minutes/week), then exercise was insufficient; and (3) if participants reported no moderate-intensity and highintensity exercise, then they were defined as inactive.

### Definitions of Hypertension and aTRH

According to NHANES, patients with hypertension were defined as meeting one of the following criteria [27]: (a) had been told that they had hypertension or had taken and were taking antihypertensive medication; (b) had a mean systolic blood pressure on the NHANES test that was more than or equal to 130 mm Hg or a mean diastolic blood pressure that was greater than or equal to 80 mm Hg (Participants sat quietly for 5 minutes and then had their blood pressure measured 3 times, taking the average of the 2^nd^ and 3^rd^ measurements.).

aTRH is usually defined as uncontrolled blood pressure (systolic blood pressure ≥ 140 mmHg or diastolic blood pressure ≥ 90 mmHg) despite receiving ≥ 3 different antihypertensive medications or requiring ≥ 4 antihypertensive drugs to control blood pressure [5].

### Covariates

The following categorical variables were included in the study as covariates: gender, age, race, BMI, PIR, smoking, alcohol drinking, eGFR, ALT, AST, and diabetes. Race was categorized as Mexican American, other Hispanic, non-Hispanic white, non-Hispanic black, and other race. PIR was calculated by dividing total household income by the annual poverty threshold determined by the U.S. Census Bureau based on household size; PIR was categorized into 3 categories: low (≤ 1.3), medium (1.3–3.5), and high (> 3.5), as recommended by the NHANES [28]. BMI (measured weight/height^2^) was categorized into 3 groups of < 25 kg/m^2^, 25–30 kg/m^2^ and ≥ 30 kg/m^2^ [29]. “Now smoking” is defined as having smoked more than 100 cigarettes in a lifetime and currently smoking every day or sometimes; “Former smoking” is defined as having smoked more than 100 cigarettes in a lifetime but not currently smoking; “Never smoking” is someone who has smoked less than 100 cigarettes in their lifetime [30]. According to the NHANES Alcohol Use Questionnaire, “In any one year, have you had at least a 12 oz. beer, a 5 oz. glass of wine, or one and half ounces of liquor” defines whether or not to drink alcohol [31]. Diabetes mellitus was defined in those who met one of the following criteria: a) physician notification of diabetes mellitus; b) taking hypoglycemic medication; c) glycosylated hemoglobin > 6.5 (%); and d) fasting blood glucose > 126 (mg/dL) [32]. The eGFR was calculated by the Chronic Kidney Disease Epidemiology Collaboration (CKD-EPI) equation, eGFR = 141 × min(Scr/κ, 1) α × max(Scr/κ, 1)^-1.209^ × 0.993^Age^ × 1.018 [if female] × 1.159 [if black]; where Scr is the concentration of creatinine, and κ values was taken according to gender, 0.9 for males and 0.7 for females, and the α value was taken according to gender, -0.411 for males and -0.329 for females [33]. AST and ALT values were obtained from the NHANES laboratory data file and were measured using a RocheCobas 6000 (c501 module) Analyzer (Roche Diagnostics, Indianapolis, IN, USA) [34].

### Survival Data

All-cause mortality refers to death from any cause. NHANES Public Link Mortality File was utilized to obtain the cause of death. This file contains mortality follow-up data for NHANES participants as of May 13, 2022, obtained through the National Death Index (NDI) linkage: https://ftp.cdc.gov/pub/Health_Statistics/NCHS/datalinkage/linked_mortality/.

### Statistical Analysis

Analysis was conducted using R software (version 4.2.1). The ‘tableone’ package [35] was used to draw a baseline table including participants with hypertension. Group participants based on whether they have aTRH, and report sample size and proportion for categorical variables, and the mean and standard deviation for continuous variables (n is unweighted, n(%), mean and Standard Deviation (SD) are weighted).

A weighted logistic regression model of association between recreational aerobic activity and aTRH was constructed using the “survey” package [36], with odds ratio (OR) and 95% confidence interval (95% CI) to quantify relationship. Regression model was tested by gradually adjusting for potential confounders. Model I, unadjusted, was designed to demonstrate the original association between LTAPA and aTRH, contributing to a preliminary understanding of the relationship between the variables. Model II, partially adjusted, adjusted for gender, age, race, PIR, alcohol drinking, smoking, and BMI. These variables are known risk factors for hypertension and cardiovascular disease and may be associated with participation in LTAPA. Model III, fully adjusted, further adjusted for diabetes mellitus, eGFR, AST, and ALT, which may influence blood pressure regulation through metabolic pathways or liver function, based on Model II to more fully assess the independent relationship between LTAPA and aTRH. In addition, subgroup analyses were conducted based on gender and alcohol drinking to explore the association between the two and whether there is interaction in different groups, and interaction term tests were performed. Weighted Cox regression models were utilized to analyze association between LTAPA and the all-cause mortality rate of aTRH patients. Weighted K-M curves were constructed using ‘jskm’ [37], and log-rank tests were utilized to test the differences in survival between groups. In this study, a two-sided p-value < 0.05 was considered statistically significant.

## RESULTS

### Baseline Characteristics of Participants

Baseline characteristics of overall study population and subgroups included in this study are shown in Table 1. 6,705 hypertensive patients were included, with 6,292 non-aTRH patients and 413 aTRH patients, with balanced gender ratios in each group. Baseline results showed significant differences in age, race, PIR, BMI, number of cases of diabetes, and eGFR values between aTRH patients and non-aTRH patients (p < 0.05). Among them, aTRH patients were older (68.15 vs. 61.75), and the number of obese (BMI > 30 kg/m^2^) and diabetes cases were higher; however, the eGFR values of aTRH patients were significantly lower than those of non-aTRH patients (68.26 mL/min/1.73 m^2^ vs 79.47 mL/min/1.73 m^2^, p < 0.001). It is worth noting that the weekly LTAPA time of aTRH patients was significantly lower than that of non-aTRH patients (76.15 vs. 103.16, p = 0.016).

**TABLE 1:** Characteristics of NHANES participants with hypertension between 2007–2018

Characters	Total	Non-Resistant hypertension	Resistant hypertension	P Value
**Overall**	6705	6292 (95.1)	413 (4.9)

**Gender, N (%)**				0.992
**Female**	3452 (52.8)	3245 (52.8)	207 (52.8)	
**Male**	3253 (47.2)	3047 (47.2)	206 (47.2)	

**Age, (years), mean (SD)**	62.06 (12.63)	61.75 (12.62)	68.15 (11.19)	< 0.001

**Race, N (%)**				< 0.001
**Mexican American**	713 (4.6)	676 (4.6)	37 (4.5)	
**Other Hispanic**	592 (3.9)	564 (3.9)	28 (3.6)	
**Non-Hispanic White**	3108 (73.0)	2937 (73.4)	171 (65.4)	
**Non-Hispanic Black**	1743 (12.8)	1590 (12.3)	153 (22.2)	
**Other race**	549 (5.8)	525 (5.8)	24 (4.4)	

**PIR, N (%)**				0.003
≤ **1.3**	2058 (19.5)	1913 (19.3)	145 (24.1)	
**1.3–3.5**	2716 (38.7)	2551 (38.5)	165 (44.1)	
**> 3.5**	1931 (41.8)	1828 (42.3)	103 (31.8)	

**BMI (kg/m^2^), N (%)**				0.032
**< 25**	1090 (14.8)	1044 (14.9)	46 (11.3)	
**25–30**	2155 (31.5)	2041 (31.8)	114 (26.3)	
≥ **30**	3460 (53.7)	3207 (53.2)	253 (62.4)	

**Smoking, N (%)**				0.232
**Never smoking**	3347 (49.8)	3145 (49.8)	202 (50.0)	
**Former smoking**	2347 (36.1)	2190 (35.9)	157 (39.2)	
**Now Smoking**	1011 (14.1)	957 (14.3)	54 (10.8)	

**Alcohol drinking, N (%)**				0.123
**No**	1989 (24.3)	1857 (24.1)	132 (28.3)	
**Yes**	4716 (75.7)	4435 (75.9)	281 (71.7)	

**Diabetes, N (%)**				< 0.001
**No**	4123 (66.8)	3927 (67.6)	196 (49.9)	
**Yes**	2582 (33.2)	2365 (32.4)	217 (50.1)	

**eGFR (mL/min/1.73 m^2^), mean (SD)**	78.93 (21.95)	79.47 (21.69)	68.26 (24.12)	< 0.001

**AST (U/L), mean (SD)**	26.04 (16.44)	26.06 (16.69)	25.67 (10.42)	0.663

**ALT (U/L), mean (SD)**	25.18 (22.82)	25.26 (23.23)	23.6 (12.51)	0.128

**Leisure-time aerobic physical activity time (min/week), mean (SD)**	101.85 (175.81)	103.16 (176.47)	76.15 (160.59)	0.016

Note: n is not weighted and n(%), Mean and SD are weight-adjusted. PIR, Poverty-to-income ratio; BMI = body mass index; eGFR = estimated glomerular filtration rate; AST = aspartate aminotransferase; ALT = alanine aminotransferase

### Association of LTAPA Time with aTRH

To analyze the possible association of LTAPA time with aTRH, we constructed three models using weighted Logistics model to analyze the association between weekly LTAPA time and aTRH (Table.2). In the continuous Model I without adjusting confounding variables, increased LTAPA time was associated with a reduced risk of developing aTRH (OR = 0.939, 95%CI [0.884,0.999], p = 0.045). In the adjusted Model II and III, the corresponding risks decreased. However, this association was not significant in models after adjusting for confounding variables, reflecting the confounding effects of these variables.

**TABLE 2:** Associations between leisure-time aerobic physical activity time and OR (95% confidence intervals) for resistant hypertension

Leisure-time physical activity time (min/week)	Model I	Model II	Model III
**Continuous**	0.939 (0.884, 0.999), 0.045	0.973 (0.916, 1.033), 0.365	0.996 (0.981, 1.011), 0.578

**Categorical**			
**0**	Ref.	Ref.	Ref.
**0–90**	0.892 (0.555, 1.432), 0.632	1.010 (0.628, 1.624), 0.966	1.066 (0.669, 1.700), 0.784
≥ **90**	0.676 (0.496, 0.923), 0.014	0.844 (0.622, 1.146), 0.274	0.896 (0.660, 1.216), 0.475
*P*_trend_	0.015	0.290	0.508

Model I was unadjusted. Model II: adjust for gender, age, race, PIR, alcohol, smoke and BMI. Model III: model II plus adjustment for diabetes, eGFR, AST and ALT.

Weighted tertile groups were created based on LTAPA time, with the group of 0 minutes of aerobic exercise per week as the reference. The association between weekly aerobic exercise time and aTRH was further analyzed. The results showed that in Model I without adjusting for covariates, compared to the group with 0 minutes of aerobic exercise per week, participants with ≥ 90 minutes of aerobic exercise per week were associated with the decreased risk of aTRH significantly (OR = 0.676, 95% CI [0.496, 0.923], p = 0.014). In Model II adjusting for some covariates (OR = 0.844, 95% CI [0.622, 1.146]) and Model III adjusting for all covariates (OR = 0.896, 95% CI [0.660, 1.216]), the risk of aTRH in the group with ≥ 90 minutes of aerobic exercise per week decreased but not significantly (p > 0.05). P_trend_ represents the trend change of LTAPA time across weighted tertile groups. A significant trend change was only observed in Model I (p = 0.015), indicating that in the model without adjusting for confounding variables, increasing LTAPA time was associated with the decreased risk of aTRH.

### Stratified Analysis

The results of the interaction term test for subgroup analysis showed a significant interaction by gender (p for interaction = 0.045 < 0.05), therefore, we further explored association of LTAPA time with aTRH when stratified by gender. The results are shown in Table.3, Model III showed that association of LTAPA time with aTRH prevalence was more significant in females (Female. OR = 0.896, 95%CI [0.811, 0.989], p = 0.029), and this association was not significant in males (Male: OR = 1.027, 95% CI [0.961, 1.099], p = 0.422).

**TABLE 3:** Associations between leisure-time aerobic physical activity time and OR (95% confidence intervals) for resistant hypertension group by gender

Leisure-time aerobic physical activity time	Model I	Model II	Model III	P for interaction
**Gender**				0.045

**Female**	0.836 (0.747, 0.936), 0.002	0.882 (0.799, 0.973), 0.013	0.896 (0.811, 0.989), 0.029

**Male**	0.990 (0.922, 1.062), 0.775	1.017 (0.949, 1.089), 0.631	1.027 (0.961, 1.099), 0.422

Model I was unadjusted. Model II: adjust for age, race, PIR, alcohol, smoke and BMI. Model III: model II plus adjustment for diabetes, eGFR, aspartate aminotransferase and alanine aminotransferase.

Given that the relationship between alcohol drinking and blood pressure has been a widely studied topic [38], we further conducted a stratified analysis based on whether female patients drink alcohol. As shown in Table 4, we found that in female patients who did not drink alcohol, the fully adjusted Model III indicated a significant relationship between LTAPA time and aTRH (OR = 0.843, 95%CI [0.714, 0.994], p = 0.043). In female patients who drink alcohol, this association was not significant (OR = 0.911, 95% CI [0.809, 1.027], p = 0.126).

**TABLE 4:** Associations between leisure-time aerobic physical activity time and OR (95% confidence intervals) for female participants with resistant hypertension group by alcohol drinking

Leisure-time aerobic physical activity time	Model I	Model II	Model III
**Alcohol drinking**			

**No**	0.789 (0.661, 0.942), 0.009	0.832 (0.704, 0.983), 0.031	0.843 (0.714, 0.994), 0.043
**Yes**	0.859 (0.752, 0.981), 0.025	0.897 (0.798, 1.008), 0.068	0.911 (0.809, 1.027), 0.126

Model I was unadjusted. Model II: adjust for age, race, PIR, smoke and BMI. Model III: model II plus adjustment for diabetes, eGFR, aspartate aminotransferase and alanine aminotransferase.

### Survival Analysis

Relationship between LTAPA time and all-cause mortality rate of aTRH patients was analyzed through weighted Cox regression analysis. As listed in Table 5, in the continuous models Model I, II, and III, an increase in LTAPA time was associated with significant reduction of all-cause mortality risk of aTRH patients (HR < 1, p < 0.05). Furthermore, aTRH patients were divided into inactive group (0 min/week), insufficient group (0–150 min/week), and sufficient exercise group (≥ 150 min/week) based on LTAPA time, and the all-cause mortality risk of each group was compared. Compared to inactive group, Model I, II, and III all showed a significant decrease in risk of all-cause mortality in group with insufficient exercise (HR < 1, p < 0.05). The group with sufficient exercise only showed a significant reduction in risk of all-cause mortality in Model I (HR < 1, p < 0.05). In addition, we also conducted a trend test, and the results showed that all Cox regression models exhibited a trend, indicating that overall, as the LTAPA time increased, risk of all-cause mortality in aTRH patients decreased (P_trend_ < 0.05).

**TABLE 5:** Cox regression analysis of leisure-time aerobic physical activity time with all-cause mortality in participants with resistant hypertension

Leisure-time aerobic physical activity time (min/week)	Model I	Model II	Model III
**Continuous**	0.827 (0.733, 0.932), 0.002	0.877 (0.797, 0.966), 0.008	0.900 (0.831, 0.976), 0.010

**Categorical**			
**Inactive (0)**	Ref.	Ref.	Ref.
**Insufficient (0–150)**	0.262 (0.145, 0.472) < 0.001	0.362 (0.189, 0.695), 0.002	0.444 (0.230, 0.854), 0.015
**Sufficient (≥ 150)**	0.447 (0.263, 0.762), 0.003	0.555 (0.296, 1.040), 0.066	0.635 (0.362, 1.116), 0.114

*P*_trend_	0.001	0.018	0.046

Model I was unadjusted. Model II: adjust for gender, age, race, PIR, alcohol, smoke and BMI. Model III: model II plus adjustment for diabetes, eGFR, aspartate aminotransferase and alanine aminotransferase.

**FIG. 2:**
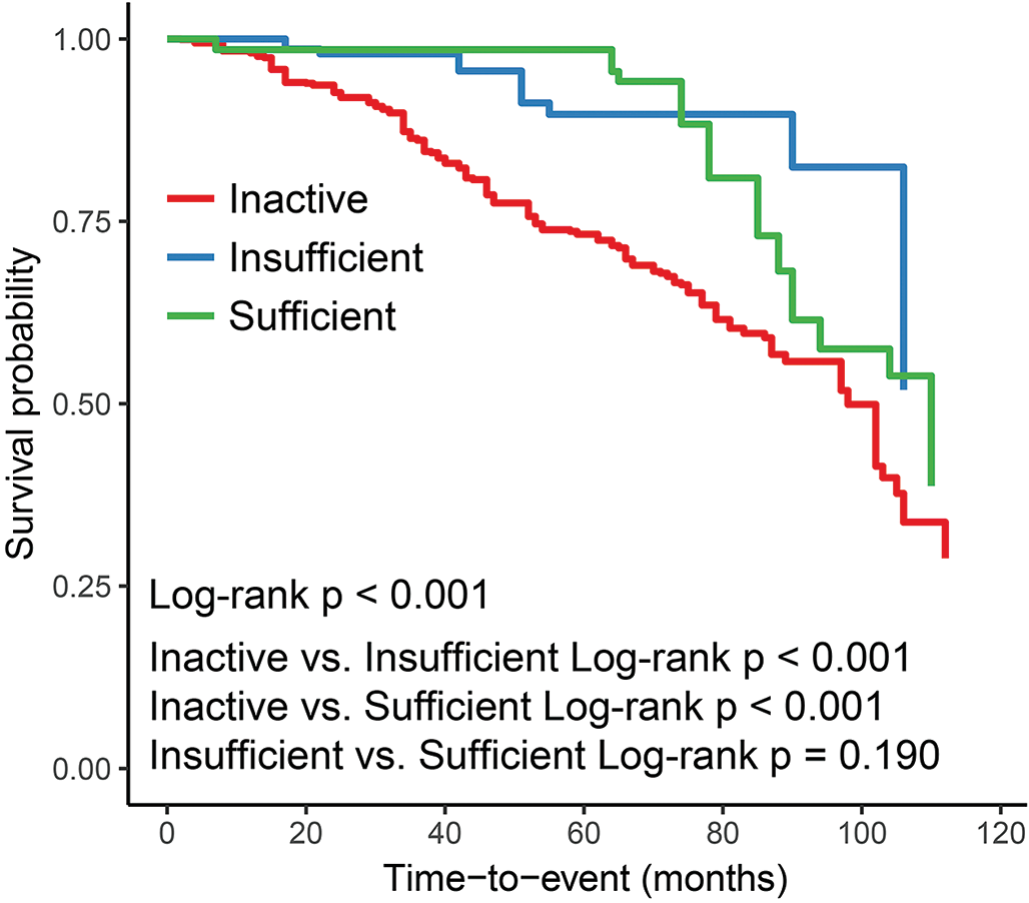
Kaplan-Meier Survival Curves for All-Cause Mortality by Leisure-Time Aerobic Physical Activity Time

After adjusting for all confounding factors, we plotted Kaplan-Meier survival curve for all-cause mortality (Figure 2). Compared to aTRH patients in inactive group, survival time of aTRH patients in the group with insufficient exercise and group with sufficient exercise was significantly prolonged (Log-rank p < 0.001), while no significant difference was seen between the group with insufficient exercise and the group with sufficient exercise (Log-rank p = 0.190).

### DISCUSSION

This study aimed to delineate relationship between LTAPA and aTRH. The results indicated that increasing LTAPA time was associated with significant reduction of the risk of aTRH incidence (p < 0.05). This association was more significant in non-drinking women. According to a weighted Cox regression analysis, significant negative association between LTAPA time and risk of all-cause mortality was observed and the risk tended to decline as LTAPA length increased. Kaplan-Meier survival curves also demonstrated that compared to the inactive group, patients in the insufficient exercise and sufficient exercise groups had significantly prolonged survival time (Log-rank p < 0.001).

In this study, the proportion of obese individuals among aTRH patients was 62.4% vs. 53.2%) and the proportion of diabetes patients (50.1% vs. 32.4%) were significantly higher in aTRH patients than in non-aTRH patients. The eGFR of aTRH patients was significantly lower than that of non-aTRH patients (68.26 mL/min/1.73 m^2^ vs. 79.47 mL/min/1.73 m^2^). This is consistent with previous studies, as Kaczmarski et al. revealed that aTRH patients had a higher prevalence of diabetes compared to non-aTRH patients (39.5% vs. 24.5%), and the eGFR of aTRH patients was lower than non-aTRH patients (68 mL/min/1.73 m^2^ vs. 82 mL/min/1.73 m^2^) [39].

The reason for the higher proportion of diabetes patients in aTRH may be closely related to the reduced insulin secretion and insulin resistance in diabetes patients. Blood vessels are an important target organ of insulin, and insulin can stimulate endothelial cells to produce NO (Nitric Oxide) (NO is one of the important endogenous blood pressure regulators) [40, 41]. Insulin resistance can cause endothelial dysfunction [40, 42]. Therefore, diabetes patients may be more prone to developing hypertension. eGFR is an important indicator of renal function, and a decrease in eGFR indicates impaired kidney function. Muntner et al. reported that aTRH patients have a higher risk of end-stage renal disease [5].

Exercise that the body does when there is a sufficient supply of oxygen is referred to as aerobic exercise. Long length, rhythmic, and moderate intensity are the hallmarks of aerobic exercise. Walking quickly, running, swimming, cycling, calisthenics, and jumping rope are examples of common aerobic workouts. After assessing the findings of 34 meta-analyses, the EAPC and ESC Council on Hypertension produced a consensus paper in March 2021 on customized exercise prescription for the prevention and treatment of hypertension. This document advocated aerobic exercise as the primary option of physical activity for patients with hypertension and can help lower patients’ mean systolic and diastolic blood pressure by 4.9 to 12.0 mmHg and 3.4 to 5.8 mmHg, respectively [43]. Endothelial dysfunction is one of the characteristic abnormal changes in hypertension, and the NO-mediated diastolic vascular pathway is thought to be important for blood pressure regulation [44]. Exercise can improve endothelium-dependent vasodilation and increase vascular NO generation [45, 46]. Aerobic exercise can effectively reduce ROS in oxidative stress-associated diseases like hypertension, and reduction of ROS is crucial for protecting endothelial cell structure and NO activity [47]. Li et al.’s study also suggests that long-term aerobic exercise can improve insulin signaling, thereby restoring vascular dilation and lowering blood pressure [48]. Zhang et al.’s study also shows that engaging in moderate-intensity physical activity for more than 300 minutes per week can reduce the incidence of RH by 17% [15]. Physical activity in this study refers to activities related to work, transportation, and leisure, and it did not specify whether aerobic exercise alone can reduce the incidence of RH. Therefore, this study further explored association of LTAPA with RH and obtained positive results. In addition, our study also indicated that an increase in LTAPA time was beneficial in reducing the risk of all-cause mortality and prolonging survival time in patients with aTRH. Therefore, for patients with hypertension, the importance of LTAPA in disease control should be emphasized, and it is actively advocated to strengthen blood pressure management by changing lifestyle to reduce risk of aTRH and mortality.

There are gender differences in RH in terms of demographics, lifestyle, and clinical aspects. For example, women account for a higher proportion of patients with RH [49, 50] and the control rate of blood pressure in women is lower than that in men [49]; while male patients are often younger than female patients, visceral organ damage is more common, and risk of cardiovascular events is higher [51]. Therefore, it is necessary to explore association of LTAPA time with aTRH at the gender level. It is worth noting that our study suggested that association of LTAPA time with aTRH was more significant in women, especially in non-drinking female patients. This research result may be related to the different regulatory mechanisms of vascular function and blood pressure in women and men. For example, with aging and obesity, women have a greater increase in sympathetic nerve activity than men, and this increase in sympathetic nerve activity is mainly reflected in higher blood pressure; women have lower reflex sensitivity of pressure receptors than men, resulting in a lower rate of blood pressure control in women than in men; and lower estrogen levels in postmenopausal women have been linked to activation of renin-angiotensin-aldosterone system, which elevates blood pressure by releasing angiotensin II and angiotensin III to constrict systemic vasculature, and with activation of sympathetic nervous system and reduced vascular NO bioavailability [52]. The mechanisms by which aerobic exercise reduces blood pressure include enhancing sensitization of pressure receptors [53, 54], reducing sympathetic nerve activity to lower blood pressure and thus reducing vasoconstrictor release [19, 53]. Similarly, alcohol drinking causes an increase in blood pressure, with studies reporting a 1 mmHg increase in blood pressure for every 1 g of alcohol ingested [55]. Vacca et al. stated that the adverse effects of alcohol on blood pressure can be mediated through neurohormonal mechanisms, such as by activating sympathetic nervous system causing an elevated risk of hypertension, which in turn alters sensitivity of vascular pressure receptors [38]. This suggests that alcohol drinking may reduce the effect of LTAPA on blood pressure in female patients. In conclusion, gender-specific exercise strategies should be considered, and tailored treatment strategies for hypertension patients should be developed by combining a healthy lifestyle to lower risk of mortality.

This study investigated the link between LTAPA time and aTRH by doing a cross-sectional analysis of the NHANES data from 2007 to 2018. This is a cross-sectional study with a large sample size, and our results offer evidence in favor of using non-pharmacological treatments like LTAPA to avoid aTRH. This study is constrained, though. Firstly, because of the cross-sectional design of this study, although an association between LTAPA time and the risk of aTRH prevalence was observed, it was not possible to determine whether this association was causal. We will further conduct high-quality prospective cohort studies to validate the results of this study. Furthermore, the blood pressure measurements in this study only included clinic blood pressure and did not include dynamic blood pressure monitoring, which may lead to data bias. In addition, LTAPA data in this study was self-reported by patients, and participants may not have been able to accurately recall their frequency and duration of exercise over time, which could affect the accuracy of the data. Also, the lack of objective means of validation for self-reported data may lead to bias. Finally, although this study had a large sample size, the NHANES participants were primarily U.S. adults and may not be fully representative of populations in other countries or regions.

## CONCLUSIONS

This study suggested that increased LTAPA time was significantly associated with a reduced risk of aTRH prevalence, especially in female patients who do not consume alcohol. It is recommended that future research should focus on gender differences, with an emphasis on female-specific intervention trials, to explore the effects of specifically designed exercise programs (e.g., combining aerobic exercise and strength training) on blood pressure control and cardiovascular health in female hypertensive patients. In addition, LTAPA was significantly beneficial in reducing the risk of all-cause mortality and prolonging survival time in patients with aTRH. Future studies should utilize a high-quality prospective cohort design incorporating ambulatory blood pressure monitoring and objective exercise tracking tools to validate our results and explore potential causal mechanisms. In summary, this study provides data support for LTAPA as a nonpharmacological intervention for the prevention and treatment of aTRH and emphasizes its importance in the management of hypertension.

## Data Availability

All the data within this manuscript could be gotten from corresponding author at reasonable request.
